# Indents

**Published:** 1915-01

**Authors:** 


### INDENTS.

☞ **Brief Articles of Technical or Scientific Value will receive notice and comment. Contributions invited.**

*Our Aim—A Better Individual Dentist.* "The dental profession can not successfully handle the problems now confronting it with the present type of dentists. We must develop in our school and from among ourselves a new type. There must be a new dentistry, just the same as there has been in process of development a new medicine. The new dentist must be essentially and especially a man who thinks, whose principal service to his patient is mental, not mechanical. This man must also have a broader basis for his thinking than we have today.

We are facing a problem which can not be worked out for us by someone else. It is ourselves—each one of us—that needs this development. And so it must be with the problems of today and tomorrow. A score of researchers and several million dollars can not change the dental profession to a group of men who will seriously undertake to do their part in preserving and promoting health.

The dental society which does the most good in this development is the society which promotes those plans which tend to invite, induce or compel the largest number of members to study, to think, to write and discuss those closer and finer details which go to make up every problem which confronts us."—*Bulletin Indiana State Dental Association.*

*Forsyth Dental Infirmary.* One can hardly find words to express the beauty of the building and its location on the Fenway, a short distance from Harvard University. The building is of white marble, and as one of the trustees expressed it, "There is not enough wood in the structure to make a tooth-pick."

I do not know of an improvement which could be made in any building to be erected for the same purpose. They have so many things distinctly original.

The day was perfect, which aided the dedication ceremonies. Dr. Gallie was the only representative from Illinois and I the only one from Michigan. However, there were a number of men from the Eastern States.

There is a surgical operating room with ampitheatre which is a little jewel. Besides an oral surgeon they have rhinologist, who looks after the diseases of the adenoids and tonsils. A dozen little beds close by, a corps of nurses and a diet kitchen, complete in every detail, makes a charming little hospital. Should occasion require, there is room for twenty-five or thirty beds.

There is a beautiful playroom for the children equipped with boxes of sand, an aquarium in the center of the room, blocks, etc. Special tiling has been made, decorated with nursery rhymes and pictures.

A splendid X-ray equipment, also a large lecture room with lantern, where lectures will be given to the members of the profession, students, and the public, all add to the completeness of the institution.

Suffice to say that it is necessary to see the building, its equipment and environment to fully appreciate it. Boston will now be looked upon as the Mecca for the dental profession in the field of oral hygiene.—C. H. Oakman.

*Emetine—A "Double-Barrelled" Specific.* During the last few months, we have had occasion to say a good deal in these pages about emetine; and there have been excellent reasons for the space devoted to the discussion of this drug.

When Rogers, of Calcutta, demonstrated that in the pure, crystalline alkaloid of ipecac we had a definite *cure* for tropical dysentery, one of the great scourges of the world, he ushered in an epoch in the history of medicine. From that day on, emetine took rank, as a specific, with quinine in malaria, salvarsan and mercury in syphilis, and antitoxin in diphtheria.

Nor is that all. Within the last six months, the discovery has been made by Barrett and Smith, of Philadelphia, a discovery verified by Bass and Johns, of New Orleans, that emetine is, in addition, specific for another devastating disease—pyorrhea alveolaris. All the evidence available seems to show that it is just as definitely curative in this disease as it is in dysentery.

I hope every reader of *Clinical Medicine* will read the abstract of Doctor Bass' article, which appeared in last month's issue, on page 1000, also his article on page 1106, this issue. There have also come to our attention reports of a number of cures of pyorrhea following the administration of emetine, in severe cases, which had failed to respond to other methods of treatment. For instance, we know of one New York dentist who employed this drug in a case in which pyorrhea affected virtually all the teeth in the patient's mouth. The gums were spongy, the gingeval sacs were exuding pus in large quantities, and the teeth were loosening. The patient was in very poor health, as a result of the condition of the mouth. After a few days' treatment with emetine, the clinical picture was changed for the better in a manner and degree which were simply marvelous.

There is no doubt that what was accomplished in this instance can be accomplished in thousands of others. And it should be remembered that, in curing pyorrhea, the dentist or the physician giving this treatment is undoubtedly preventing other more or less remote but crippling diseases, since it is now believed that a large percentage of the chronic arthritides (arthritis deformans, for instance) are caused by pyorrhea.

It will be seen, therefore, that emetine possesses the unique distinction of being the one remedy in the world that apparently is a specific for two separate and distinct diseases. It cures tropical dysentery, and it cures pyorrhea. In addition, its power of checking internal and external hemorrhage (especially hemoptysis) promptly has been demonstrated again and again, and the number of successes is constantly growing; and it still retains its place as one of the most valuable remedies at our disposition for the relief of the dry cough and viscid expectoration, which is so characteristic of various stages of bronchitis and pneumonia.

We can not urge upon our readers too strongly the importance of a careful re-study of this alkaloid.—Editorial in *Clinical Medicine.*

*Still Another Pellagra Theory.* In May, 1913, Alessandrini and Scala, of Rome, announced the cause and specific treatment of pellagra. Alessandrini sent me his monographs, and I am enclosing a brief resume of their conclusions. They are very definite and exact, and should be a boon to American physicians. They are as follows:

THE CAUSE OF PELLAGRA.

1. Pellagra is a chronic intoxication caused by the presence of colloidal silica in drinking water.

2. Pellagra is a disease strictly localized and contracted in districts were the water supply is in contact with an argillàceous terrain.

3. Colloidal silica makes irreversible and insoluable compounds with the proteid substance of the tissue cells.

4. This combination abstracts water from the tissues, the resulting drying giving rise to cutaneous manifestations and digestive and nervous disturbances, the classical triad of pellagra.

5. The acid intoxicating is concentrated by evaporation, hence the cutaneous manifestations on the face, neck and hands.

6. Pellagra is not affected by maize alimentation.

7. Pellagra is not due to filaria.

8. Pellagra is not communicated by the simulium fly.
9. Pellagra is not a parasitic disease.

PREVENTION AND TREATMENT.

1. Pellagra is prevented by treating water supplies and reservoirs with broken limestone.
2. The essential treatment of pellagra is alkaline.
3. Inject intramuscularly in the gluteal region 1 Cc. of a 10 per cent. solution of trisodic citrate, daily at first, later at longer intervals.
4. Treatment of the ordinary case will take from one to two months; the number of injections will be about one-half the number of days under treatment.
5. This treatment will cure pellagra without change of diet, domicile, occupation, or sanitary environment.—E. M. Perdue, Kansas City, Mo., in *Clinical Medicine.*

*Poisonous Mushrooms.* In addition to what is said in the November number of *Clinical Medicine,* page 1007, on mushroom poisoning, I want to add a word as to recognition of species.

All the species of amanita have a ring round the upper part of the stem and a cup, or volva, at the base of the stem. In *amanita phalloides* the cup is very distinct, but in *amanita muscaria* it looks like several cups, or layers. The top of the pileus is pale yellow, covered with distinct warts an eighth of an inch or more in diameter. All the species of amanita are white underneath the pileus.

By noticing these points no one need gather any of the amanitas to eat. While some of the species of amanita are reported as nonpoisonous, it is well to avoid all of them.—G. H. French, Carbondale, Ill.

---

[It is well to repeat the therapeutic and diagnostic advice given in the article to which Professor French refers. If symptoms appear early, then muscaria poisoning may be suspected, and for this, atropine is the indicated remedy. If the appearance of the symptoms is delayed (36 to 48 hours), then treatment must be symptomatic, special attention being given to excretion.—Ed.]

*How to Have Good Health.* Have good digestion.

Good teeth are necessary in order to have good digestion.

Teeth cleaning required in order to have good teeth.

Chew your food thoroughly and slowly; don't make your stomach do the mouth's work.

Exercise your teeth. Teeth decay from want of use. For that reason tough beefsteak, as well as other hard food, is good for you.

Mix saliva with your food; the stomach needs the saliva to help in digestion; your mouth is equipped with saliva glands, and there is always plenty on hand. To mix, chew food thoroughly until it is a pulp.

*The Dental Toilet.* Toothbrush, with serrated bristles, that spaces between the teeth may be reached. Teeth should be brushed up and down.

Good tooth powder or paste.

Dental floss, silk thread, which pulled between the teeth, removes particles lodged there.

Mouth wash, preferably warm, for rinsing; use after brushing teeth.

Brush teeth three times a day. On rising and particularly on retiring at night, the most important time of all, and after the mid-day meal.

---

Let the baby have the crust. Don't take it away and leave it only the soft part of the bread. The crust is its better friend, declare the dentists.

Joliet dental physicians met last night in the Commercial Club, and after following their own rules regarding the mastication of the planked white fish, and the soup and ice cream, considered the ways of the world in regard to its teeth and its ignorance and mis-information about teeth generally.

Just as the crust is good for the baby to chew on, so is the tough beefsteak and hard Swedish bread good for the adult, if he wants perfect teeth fastened solidly in his jaw.

The teeth need exercise quite as much as any other member of the body.—*Bulletin Illinois State Dental Society.*

*Locating Root Canal Openings.* Dr. Hyatt in Ottolengui's "Round Table" says he first swabs the floor of the cavity in a multi-rooted tooth with a pledget of cotton saturated with tincture of iodine. He then wipes out the excess iodine with fresh cotton but the iodine, that penetrates the canal openings remains to mark them distinctly. This makes instrumentation definite, as it locates exactly the canals.

*Sterilizing Operating Field.* Dr. Van Woort, when placing the rubber dam for root canal work, first cleanses the immediate region thoroughly with an antiseptic soap. He then paints it thoroughly with tincture of iodine. He then lubricates the hole in the rubber dam with a little carbolized vaseline. This makes the placing of the dam and clamp a sterile or non-infectious procedure. This may seem unnecessary, but it is no more care than the surgeon takes in every operative procedure. —*Items of Interest.*

*Conductive Anesthesia.* Formerly dental and oral surgery was done with much difficulty because no efficient anesthetic was available. Nitrous oxid-oxygen is not satisfactory in many cases. Ether is too complicated and unsafe in the hands of the average dentist. The introduction of the so-called "inductive anesthesia,"

has made it possible to sterilize extended regions of the oral cavity profoundly for several hours. This makes many former dental operations more comfortable, and there will undoubtedly be much use for this valuable adjunct to our surgery. Let us hope it may not be abused.

